# Cardio-Oncology Rehabilitation—Present and Future Perspectives

**DOI:** 10.3390/life12071006

**Published:** 2022-07-07

**Authors:** Boaz Elad, Manhal Habib, Oren Caspi

**Affiliations:** 1 The Department of Cardiology, Rambam Health Care Campus, Haifa 3109601, Israel; b_elad@rmc.gov.il (B.E.); m_habib@rmc.gov.il (M.H.); 2 Technion—The Ruth and Bruce Rappaport Faculty of Medicine, Haifa 3525433, Israel

**Keywords:** cardiac rehabilitation, cardiotoxicity, cardiomyopathy, exercise

## Abstract

Recent advances in cancer therapy have led to increased survival rates for cancer patients, but also allowed cardiovascular complications to become increasingly evident, with more than 40% of cancer deaths now being attributed to cardiovascular diseases. Cardiotoxicity is the most concerning cardiovascular complication, one caused mainly due to anti-cancer drugs. Among the harmful mechanisms of these drugs are DNA damage, endothelial dysfunction, and oxidative stress. Cancer patients can suffer reduced cardiorespiratory fitness as a secondary effect of anti-cancer therapies, tumor burden, and deconditioning. In the general population, regular exercise can reduce the risk of cardiovascular morbidity, mortality, and cancer. Exercise-induced modifications of gene expression result in improvements of cardiovascular parameters and an increased general fitness, influencing telomere shortening, oxidative stress, vascular function, and DNA repair mechanisms. In cancer patients, exercise training is generally safe and well-tolerated; it is associated with a 10–15% improvement in cardiorespiratory fitness and can potentially counteract the adverse effects of anti-cancer therapy. It is well known that exercise programs can benefit patients with heart disease and cancer, but little research has been conducted with cardio-oncology patients. To date, there are a limited number of effective protective treatments for preventing or reversing cardiotoxicity caused by cancer therapy. Cardiac rehabilitation has the potential to mitigate cardiotoxicity based on the benefits already proven in populations suffering from either cancer or heart diseases. Additionally, the fact that cardiotoxic harm mechanisms coincide with similar mechanisms positively affected by cardiac rehabilitation makes cardiac rehabilitation an even more plausible option for cardio-oncology patients. Due to unstable functional capacity and fluctuating immunocompetence, these patients require specially tailored exercise programs designed collaboratively by cardiologists and oncologists. As the digital era is here, with the digital world and the medical world continuously intertwining, a remote, home-based cardio-oncology rehabilitation program may be a solution for this population.

## 1. Introduction

Recent cancer treatment advances have yielded tremendous gains, leading to longer survival and better quality of life [1]. Cancer death rates in the United States decreased by 20% from 1991 (215.1 per 100,000 in the general population) to 2010 (171.8 per 100,000 in the general population), leading to millions of cancer survivors [1]. These advances are derived from early cancer diagnoses and generally improved treatments. More than 60 anti-cancer drugs have been approved by the FDA in recent years. In addition, a deeper understanding of tumor biology has led to an entire range of new, molecularly targeted drugs. The strategy of combining different types of therapy has also shown powerful results [1].

The advances in cancer therapy have led to increased survival rates of cancer patients but also allowed for short- and long-term cardiovascular complications to become evident, leading, in turn, to premature morbidity and mortality among cancer survivors [2]. In recent years, many of the deaths among cancer patients were not attributed directly to the index-cancer and more than 40% of cancer patients’ deaths were attributed to cardiovascular diseases [3].

In general, cardiovascular complications of cancer therapy can be divided into nine main categories: myocardial dysfunction and heart failure (HF); coronary artery disease (CAD); valvular disease; arrhythmias; arterial hypertension; thromboembolic disease; peripheral vascular disease and stroke; pulmonary hypertension; and pericardial complications [2].

Cancer-therapy-related cardiac dysfunction is increasingly evident within the expanding use of novel chemotherapeutic agents. This dysfunction is a serious complication that could eventually culminate in the development of life-threatening myocardial dysfunction and HF [4], and it is one of the most common causes of non-cancer deaths [3]. HF is typically difficult to treat, substantially affecting overall morbidity and mortality, and requiring long-term therapy. Myocardial dysfunction and HF, frequently described as cardiotoxicity, are the most concerning cardiovascular complications. Cardiotoxicity may involve direct effects of the cancer treatment on the function and structure of the heart, or it may be due to accelerated developments in cardiovascular diseases (CVD) [2].

Among the leading causes for cardiotoxicity are anti-cancer drugs and radiotherapy. Anthracycline-based therapy can result in irreversible cardiac damage and HF, with an incidence of 5–48%, in a dose-dependent manner [2]. The mechanisms of 5-fluorouracil induced myocardial ischemia are multifactorial and include coronary vasospasm and endothelial injury. Other conventional chemotherapies, including Cyclophosphamide, Cisplatin, Ifosfamide, and Taxanes can lead to cardiotoxicities as well. These severe adverse consequences of therapy are particularly relevant for young cancer survivors who display increased rates of cardiovascular diseases, even several decades after therapy [2]. Immunotherapies and targeted therapies, such as Trastuzumab and other therapies targeting anti-human epidermal growth factor receptor 2, may cause cardiac dysfunction as well, especially with concomitant or previous use of anthracyclines. Vascular endothelial growth factor inhibitors can cause both reversible and irreversible cardiac side effects (particularly when used with or after conventional chemotherapies) and arterial hypertension, potentially affecting cardiac function. Potent inhibitors of BCR-ABL have also demonstrated an association with cardiovascular events. Proteasome inhibitors for treatment of multiple myeloma can potentially cause cardiac dysfunction as well. Among survivors exposed to radiotherapy, the risk of HF was increased 4.9-fold, and marked interstitial myocardial fibrosis is common in radiotherapy-induced cardiotoxicity. HF may also be aggravated by concomitant radiation-induced valvular heart disease and coronary artery disease (CAD) [2].

Risk factors for cardiotoxicity include current HF, asymptomatic left ventricle dysfunction (ejection fracture less than 50%), CAD, valvular heart disease, hypertensive heart or left ventricle hypertrophy, cardiomyopathy, arrhythmias, prior cardiotoxicity (anthracyclines, mediastinal radiotherapy, or chest radiotherapy), age of less than 18 years old or more than 50 years old, family history of CAD, and traditional cardiovascular co-morbidities and lifestyle risk factors, such as hypertension, diabetes, and hyperlipidemia [2].

## 2. Non-Cardiac Physical Deconditioning in Cancer Patients

Most cancer patients and cancer survivors will experience compromised physical and cognitive functioning due to cancer treatments and their side effects, resulting in a negative impact on their quality of life and survival rates [5]. Reduced cardiorespiratory fitness (CRF) in cancer patients is multifactorial. Cancer patients are often older than the general population and commonly present with pulmonary, cardiovascular, and/or musculoskeletal complications. Treatment with anti-cancer therapies, combined with tumor burden, can simultaneously impact components in the oxygen cascade, including the pulmonary, cardiac, hematological, vascular, and musculoskeletal systems. Indirect effects of physical inactivity (deconditioning) will further contribute to reduced CRF [6]. These patients also develop multiple medical conditions, often related to the late and long-term effects of their cancer treatment, as well as conditions related to premature aging [7]. Several studies following long-term cancer survivors have shown significant impairment in activities, physical limitations [8,9,10], and decreased exercise capacity in different cancer populations, including chemotherapy-treated and post-surgical populations [11,12,13].

The main risk factors for physical deconditioning in cancer patients include the attributes of female gender, lower income and education, central nervous system and bone cancers, brain radiotherapy, and premature gonadal failure (caused by androgen deprivation, alkylating agents, and pelvic irradiation) [11].

## 3. Mechanisms of Cardiotoxicity

**Figure 1:**
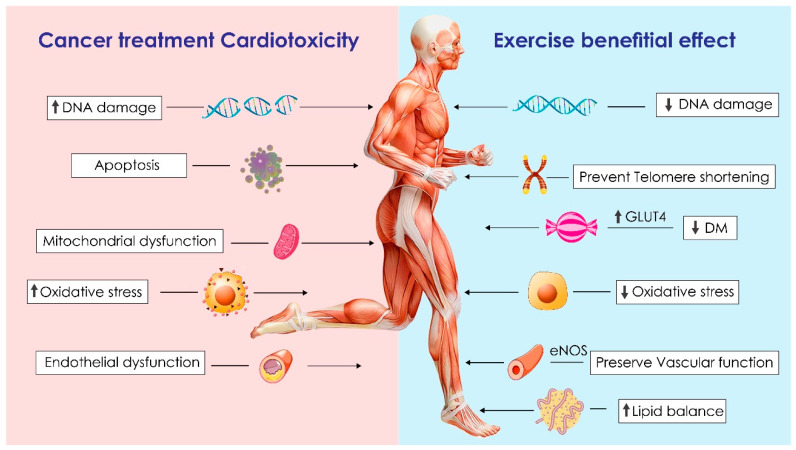
Common mechanisms mediating cardiotoxicity and positively affected by exercise. DM: Diabetes Mellitus.

There are several theories regarding the different mechanisms for cancer treatment cardiotoxicity. Contemporary treatment methods are effective in treating different modalities of cancers; however, these reagents act through interference with DNA replication or by preventing DNA repair, causing endothelial dysfunction, generating reactive oxygen species, or eliciting non-specific immune responses [14]. Anthracyclines have become a central chemotherapeutic treatment option because of their high efficacy [15]. Anthracycline cardiotoxicity is estimated to affect 9% of cancer patients treated with anthracyclines [16]. The mechanism is believed to be multifactorial. The two main hypotheses are: (1) oxidative stress, which, in the presence of iron, generates reactive oxygen species that cause lipid peroxidation of the cell membrane, leading to damage of the cardiomyocytes [17,18,19]; and (2) inhibition of topoisomerase IIβ (TOPIIβ), which is active in quiescent nonproliferating cardiomyocytes. The inhibition of TOPIIβ can result in the activation of cell death pathways and inhibition of mitochondrial biogenesis [15,20]. Another suggested mechanism is the dysregulation of cardiomyocyte autophagy [21]. Anthracycline toxicity is dose dependent. Evidence suggests cardiotoxicity occurrences of 10%, 16%, 32%, and 65% at cumulative doxorubicin doses of 250, 300, 400, and 550 mg/m^2^, respectively [22]. Trastuzumab, functioning as an inhibitor of ErbB2 receptor, interrupts ErbB4/ErbB2 heterodimerization, leading to subsequent mitochondrial energy dysfunction and apoptosis [23,24]. In addition, trastuzumab activates the autophagy-inhibitory Erk/mTOR/Ulk 1 signaling cascade, causing cardiomyocyte apoptosis [25]. 5-fluorouracil (5-FU) can lead to coronary vasospasm through Protein kinase C and Endothelin-I [26,27], direct myocardial injury through alpha-fluoro-beta-alanine (FBAL) (which is a breakdown product of 5-FU [28]), vascular endothelial dysfunction through direct toxic effects, and excess production of reactive oxygen species [29]. Moreover, mitochondrial dysfunction and apoptosis of cardiomyocytes are believed to play an important role in 5-FU induced cardiotoxicity [30]. Cisplatin can induce nuclear and mitochondrial DNA damage [31], as well as the augmentation of oxidative stress and mitochondrial dysfunction [31,32]. Metabolites of cyclophosphamide are responsible for cardiotoxicity due to depletion of antioxidants/ATP level, altered contractility, damaged endothelium, and enhanced pro-inflammatory/pro-apoptotic activities [33]. Tyrosine kinase inhibitor-induced cardiac injury is mediated by oxidative stress, mitochondrial damage, and apoptosis of cardiomyocytes [34]. Immune checkpoint inhibitors, including anti-PD-1, anti-PD-L1, and CTLA-4 blockade, can cause cardiotoxicity as a result of immune inflammation and oxidative stress [30,35,36]. Figure 1 summarizes mechanisms mediating the cardiotoxicity of selected cancer therapeutics. Table 1 details some of the main mechanisms involved in common cancer therapeutics-related cardiotoxicity.

## 4. Therapies for Prevention of Cardiotoxicity

Drugs such as Dexrazoxane have shown a significant protective effect, but their use is limited, due to their high-cost and the concern about a potential reduction in anti-cancer efficacy and a risk of developing secondary tumors [4]. Apart from Dexrazoxane, the field has thus far focused on utilizing drugs aimed at mitigating maladaptive neurohormonal activation and preventing further deterioration of cardiac function. Conventional HF drugs have demonstrated limited benefit in ameliorating the cardiotoxic damage of chemotherapy. The 2016 ESC guidelines state that the benefits from preventive treatment with ACE inhibitors, ARBs, or beta-blocker therapy remain controversial, and no recommendation can be made at this time [2,39]. Several preliminary studies on animal models found Metformin to exert anti-cancer and cardio-protective properties, although more studies are necessary [40].

## 5. Exercise Influence on the Cardiovascular System

Regular exercise, in which moderate-to-vigorous intensity is achieved, leads to a reduction in the risks of all-cause mortality, CVD, hypertension, stroke, metabolic syndrome, diabetes, and cancer. Individuals who sustain high exercise levels tend to live longer and have lower mortality rates for both CVD and cancer, compared with the general population [41]. Exercise substantially alters the expression of genes in the human genome. Exercise-induced modifications of gene expression result in rapid, yet transient, improvements in cardiovascular parameters and general fitness [41]. Exercise may prevent telomere shortening (modifying senescence, apoptosis, and oncogenic transformation of cells), affect oxidative stress and vascular function, and induce the upregulation of DNA repair mechanisms [41,42].

Exercise-induced oxidative stress continues to be a controversial topic. In theory, a moderate level of reactive oxygen species (ROS) production during exercise promotes positive physiological adaptation in the active skeletal muscles (e.g., mitochondrial biogenesis, synthesis of antioxidant enzymes, and production of stress proteins), whereas high levels of ROS production result in damage to macromolecular structures (e.g., proteins, lipids, and DNA). The increased risk of CVD occurrence is partially attributed to the development of vascular endothelial dysfunction, while regular endurance exercise is effective for maintaining overall vascular regularity and can prevent a loss of endothelium-dependent vasodilation. It has been shown that training individuals have significantly higher endothelial NOS (eNOS) expression and phospho-eNOS levels, superoxide dismutase, and an improved vascular antioxidant capacity, which was correlated with endothelial function [41]. Past studies have revealed that either endurance exercise or interval training increased the antioxidant capacity of both cardiac and skeletal muscle myocytes. Both short-term (measured in days) and long-term (3 month) endurance exercise training increase antioxidant enzyme activities in the trained muscles and eliminate the contraction-induced oxidative stress that can result from an acute bout of exercise [42]. The reduction in oxidative stress has the potential to reduce the risk of cancer as well. A large epidemiological study involving 1.44 million patients concluded that regular physical activity reduced the risk of 13 different types of cancer [43]. Exercise has been shown to reduce the risk of recurrence of tumor growth as well. The molecular mechanisms responsible for exercise-induced protection against cancer remain unclear, but an upregulation of antioxidant gene expression has been postulated to be a contributing factor [42]. Figure 1 summarizes mechanisms mediating cardiotoxicity, including the beneficial effects of exercise.

## 6. Rehabilitation for Cancer Patients

The main aims of rehabilitation for cancer patients are to regain physical activity, prevent frailty, and improve aerobic abilities. Aerobic exercise training has been widely established to be one of the most-effective therapies to improve CRF. It improves the reserve capacity of the components of oxygen transport and use, which leads to favorable improvements in VO_2peak_ [6].

Randomized trials indicate that exercise training in cancer patients, following traditional exercise prescription guidelines, is safe and well-tolerated during various conventional therapeutic modalities and is associated with 10–15% improvement in different measures of CRF. Exercise training can negate the adverse effects of therapy upon CRF [6]. Several studies in recent years have found that exercise may attenuate cancer-treatment-induced declines in CRF and improve CRF after the completion of cancer therapy in different cancer populations; the studies also demonstrated the benefit of rehabilitation in high-risk patients (e.g., frail patients, elderly patients, and those undergoing complex surgery) [7]. A meta-analysis of 27 randomized clinical trials of exercise training in cancer patients after the completion of adjuvant therapy showed significantly increased CRF compared with usual care (VO2 peak weighted mean differences, 2.45 mL O_2_·kg^−1^·min^−1^ [95% CI, 1.71–3.19]) [44]. In addition, past trials have even shown correlation with improved cardiovascular outcomes and reduced mortality [44,45,46], although most of those studies are retrospective and include meta-analysis, whereas other trials have found no benefit. Although studies of the effect of exercise on cardiovascular disease outcomes in cancer survivors have focused primarily on CRF, these studies possess a significant clinical importance, because CRF is associated with short- and long-term treatment-related toxicities, symptom burden, and all-cause and cancer-specific mortality [47]. A scientific statement from the American Heart Association in 2019 urged the development of a comprehensive model, as a part of a cardio-oncology rehabilitation program, to identify cancer patients at high risk of CVD or those who have developed cardiotoxicity related to cancer therapies, and to use the multimodality approach of cardiac rehabilitation to prevent or mitigate cardiovascular events [47]. However, there are several important limitations for the participation of cancer patients in rehabilitation programs. Due to treatment side effects, as well as fever and neutropenia during nadir, participation in standard rehabilitation programs at hospital or clinic settings might expose them to various risks and higher rates of complications. In addition, due to changing hemoglobin levels and anemia, it might be difficult to determine and adjust pulse range as an indicator for exercise intensity level. Table 2 summarizes the trial results of the main studies on rehabilitation for cancer patients.

## 7. Cardiac Rehabilitation in Cardiovascular Patients

The main goals of cardiac rehabilitation are to prevent major adverse cardiovascular events, to improve VO_2_max, and to prevent mortality. Regular physical activity is an important component of therapy for most cardiovascular diseases (CVD) and is associated with reduced cardiovascular and all-cause mortality. Current guidelines strongly recommend (Class 1, level of evidence A recommendation) that patients with CVD risk factors and an established cardiac disease, including ischemic heart disease (IHD) and HF, should perform a minimum of 150 min of moderate intensity endurance exercise training weekly. Exercise-based cardiac rehabilitation (exCR) reduces cardiac mortality and hospital readmission and improves exercise tolerance and quality of life in different CVD patients [63].

In HF patients, exercise can improve quality of life and fitness. In HF with reduced ejection fraction (HFrEF), different studies have found that exCR improves exercise capacity and quality of life. In the HF-ACTION trial, 2331 patients were randomly assigned to either a monitored aerobic exercise training followed by remote-based exercise or to standard care. After three months, the exercise program group had a statistically significant improvement in the Kansas City Cardiomyopathy Questionnaire compared with the standard care group (mean 5.21 vs. 3.28, *p* < 0.001) [64]. In addition, while the trial resulted in no significant reductions in all-cause mortality or hospitalization, after correction for prognosis predictors, a significant reduction for either mortality or hospitalization in the exercise group was noted [65]. Several meta-analyses found that exCR can reduce all-cause and HF hospitalizations, especially in highly adherent patients, while the effect on mortality is uncertain [66,67]. In HF with preserved EF (HFpEF), exercise is recommended as well, as it can improve symptoms and exercise capacity. Weight reduction in these patients is extremely important and a positive additional outcome of exercise programs [63,68]. In a meta-analysis including 276 patients with HFpEF, those participating in exercise training had significantly improved cardiorespiratory fitness (mean difference of peak oxygen uptake 2.72 mL/kg per min) and quality of life as assessed by the Minnesota Living With Heart Failure score (mean difference, −3.97) when compared with the control arm [69]. In a randomized control trial including 100 older obese patients with HFpEF, caloric restriction or aerobic exercise training led to increased peak oxygen consumption compared to the control group (1.3 mL/kg per min, *p* < 0.0011 and 1.2 mL/kg per min, *p* < 0.001 respectively), with a possible additive effect attained when combining the two interventions [70]. As for HF patients with mildly reduced EF (HFmrEF), present data are scarce, but it is believed that the benefits found in other HF populations should apply [68]. In a recent post hoc analysis on the effect of remote exCR implementation in HF patients, including 195 HFrEF and 381 HFmrEF or HFpEF patients, the completion of a rehabilitation program was a strong negative predictor of death or re-hospitalization in HFrEF (hazard ratio 0.10, *p* = 0.025) and HFmrEF or HFpEF (hazard ratio 0.11, *p* = 0.028) patients [71].

Although exCR programs are highly recommended, they are significantly underused. About 10–16% of IHD patients eligible for exCR participate in these programs [72], and this percentage drops to 4.3% when HF patients are involved [73]. ExCR programs are divided into traditional, center-based programs (the prevalent form) and remote, home-based programs (HBCR). One of the advantages of center-based programs is an increase in monitoring capabilities, making such programs suitable for high- and low-risk patients, the frail, and patients who have more severe disease or comorbidities [64], whereas remote programs can better suit patients’ daily routines, thereby potentially increasing their participation rate. Recently, a statement by the American Heart Association determined that new delivery strategies are urgently needed to improve participation, a finding that emphasizes HBCR’s importance [74]. Formerly, studies have found similar clinical outcomes with HBCR compared to center-based programs [74]. HBCR monitoring and communication intensities vary. The spectrum ranges from programs that rely on basic communication, such as telephone calls, to sophisticated telemedicine programs using advanced digital means, including the automatic transfer of exercise data, clinical data, and auto-analysis information to the exCR team.

## 8. Present Data

Although there is evidence for the beneficial effects of exCR on patients with CVD and on cancer patients, there are very little data on exCR for cardio-oncology patients. Several preventive studies on cancer patients before or during cardiotoxic chemotherapy found that exCR improves hemodynamics, symptoms, and functional disability, but it does not affect cardiotoxicity markers or cardiac function. Kirkham et al. studied the effect of aerobic and resistance training in 37 breast cancer patients during anthracycline therapy. The study showed changes in hemodynamic responses, such as drops in systemic vascular resistance and cardiac output, but no beneficial effect of exercise on cardiac function as assessed by different echocardiography measurements [75]. In a study of 28 breast cancer patients participating in exercise program (2 × 60 min supervised exercise sessions per week) during anthracycline treatment, Howden et al. found that exercise prevented the decline in VO_2peak_ (15 vs. 4% reduction, *p* = 0.010), but it did not prevent the decline in left ventricle ejection fraction or in the levels of troponin [76]. In another study, Kirkham et al. showed that, in breast cancer patients, 30 min of vigorous-intensity exercise 24 h prior to the first doxorubicin treatment attenuated NT-proBNP release and increased systolic function, but the study included only 24 patients [77]. Searching the literature for studies on the role of exCR in cancer patients with established HF yielded only one trial: a retrospective analysis of 90 patients with cancer who have HF. Exercise programs did not improve any clinical outcomes, including VO_2peak_, quality of life, hospitalization, and death, but the patients showed no proven association between cancer treatment and HF occurrence [78]. We could not find any trials including patients with HF secondary to cancer treatment toxicity. Cardiac rehabilitation effects are summarized in Table 3.

## 9. The Gaps

There are certain crucial gaps in the evidence.

To date, there has been no effective treatment to prevent or reverse cancer-therapy-related cardiotoxicity. ExCR cardio-oncology programs are scarce. Specifically, there is a growing need for out-of-hospital programs for cancer patients, as this population suffers intensive hospitalization periods, including an extremely high risk for acquiring infections in the hospital or the clinic, necessitating flexibility in exercise formats tailored to their cancer treatment regimen and disease stage. In addition, there are only limited data regarding exCR for cancer patients with or at risk of cardiotoxicity. The demonstrated benefit of exercise in other clinical populations suggests that exercise may also become a key feature of future programs for oncology patients at risk for cardiotoxicity, or for those who already developed this complication.

## 10. Future Direction

As exCR programs reduce morbidity and mortality in CVD and improve the fitness and quality of life of cancer patients, further research is urgently needed to elucidate these programs’ effects on cardio-oncology patients.

As these patients are extremely complex, cardiologists and oncologists should create a hybrid rehabilitation program, taking into account different aspects of this unique population. Cardiologists could evaluate for risk factors, including the risk of cardiovascular complications such as arrhythmia and HF, and adjust rehabilitation goals and monitor intensity during exercise accordingly. Oncologists could alert the team to complications and side effects associated with anti-cancer treatments, such as anemia, risk and anticipated timing for neutropenia and risk of infection.

At present, rehabilitation programs’ major drawbacks include low participation rates, a risk negatively influenced during the COVID-19 pandemic. It seems that future cardio-oncology programs will suffer similar limitations, with additional difficulties arising due to patients’ complexity and fear of contagious disease due to immune suppression. A novel approach should be sought to overcome these difficulties. One solution can include a remote, home-based cardio-oncology rehabilitation program with the potential to increase participation rates [74] while reducing the COVID-19 effect and contagion risk.

Currently, in the digital era, the digital world and the medical world continuously intertwine. With the emergence of digital telemedicine capabilities, including arrhythmia monitoring and continuous intra-cardiac measurements [79,80,81], the prospect of remote, digital cardio-oncology programs becoming dominant seems compelling and inevitable. These advanced programs possess the advantage of increased participation, while preserving safety for fragile patients by keeping them out of hospital, thereby saving manpower due to increased efficiency. Different programs should be developed for primary and secondary prevention in cardio-oncology patients, including programs targeting those with and without risk factors for cardiotoxicity, and programs before and after cardiotoxicity treatments, including anti-cancer drugs, radiotherapy, and post-surgery. As remote and digital programs become popular, data will be accumulated, and there will be a need for studies to confirm these programs’ efficacy and safety, and also revealing their effect on prevention and treatment of cardiac dysfunction, quality of life, hospitalization, and mortality.

## Figures and Tables

**Table 1:** Mechanisms of cardiotoxicity of common cancer therapeutics.

Drug Name/Class	Mechanism of Cardiotoxicity	References
Anthracyclines	Generation of excess free radicals	[17,18,19]
	Accumulation of iron in the mitochondria	[37,38]
	Top2β inhibition-breakage of DNA and induction of programmed cardiomyocyte death	[20]
	Dysregulation of cardiomyocyte autophagy	[21]
Trastuzumab	Activation of autophagy-inhibitory Erk/mTOR/Ulk1 signaling cascade	[25]
	Interruption of ErbB4/ErbB2 heterodimerization	[23,24]
Cyclophosphamide	Depletion of antioxidants/ATP level	[33]
	Endothelial damage	[33]
	Enhanced pro-inflammatory/pro-apoptotic activities	[33]
Cisplatin	Induction of nuclear and mitochondrial DNA damage	[31]
	Oxidative stress and mitochondrial dysfunction	[31,32]
5-Fluorouracil	Coronary vasospasm through Protein kinase C and Endothelin-I	[26,27]
	Direct myocardial injury: Alpha-fluoro-beta-alanine (AFBA)	[28]
	Vascular endothelial dysfunction: direct toxic effect and reactive oxygen species production	[29]
Tyrosine kinase inhibitors	Oxidative stress, mitochondrial damage, and apoptosis of cardiomyocytes	[34]
Immune checkpoint inhibitors	Immune inflammation with T-cell infiltration of the myocardium	[30,35]
	Oxidative stress	[30,35]

**Table 2:** Summary of studies evaluating exercise rehabilitation for cancer patients.

Author/Year	Patients Characteristic	Outcomes	Num of Patients	Exercise Type	Results
Scott, J.M. et al., 2018 [44]	mixed (meta analysis) cancer patients	CRF	3632	aerobic and combined aerobic and resistance therapy	increase in VO_2peak_
MacVicar, M.G. et al., 1989 [48]	breast cancer	CRF	45	aerobic interval training	significant increase in VO_2Lmax_
Segal, R. et al., 2001 [49]	breast cancer	CRF	123	self-directed or supervised exercise	increased in Short Form-36 physical functioning scale
Van Waart, H. et al., 2015 [50]	breast cancer	CRF	230	home-based and supervised exercise programs	prevention of decline in CRF, increase physical functioning
Haykowsky, M.J. et al., 2009 [51]	trastuzumab-treated breast cancer	CRF + LV remodeling	17	aerobic training	increased CRF, did not prevent LV remodeling
Segal et al., 2009 [52]	radiation treated prostate cancer	CRF, fatigue	121	resistance or aerobic training	increased CRF, decreased fatigue
Jones, L.W. et al., 2016 [53]	breast cancer	CVE	2973	leisure-time recreational exercise	decrease in CVE
Schmid, D. et al., 2014 [54]	breast and colorectal cancer (meta analysis)	mortality	49,095	physical activity	decreased mortality
Jones, L.W. et al., 2014 [45]	post-surgical prostate cancer	CRF	50	Aerobic Training	increased CRF
Scott, J.M. et al., 2018 [55]	adult survivors of childhood cancer	mortality	15,450	questionnaire-completed study on physical activity levels	decrease in all-cause mortality
Adams, S.C. et al., 2017 [46]	testicular cancer	CRF	63	aerobic interval training	increase in VO_2peak_
Pinto, B.M. et al., 2013 [56]	colorectal cancer	CRF	46	telephone counseling to support exercise	increase in exercise duration and fitness
Courneya, K.S. et al., 2003 [57]	colorectal cancer	quality of life	102	home-based exercise intervention	no change in outcome
Zhou, Y. et al., 2016 [58]	acute leukemia (meta-analysis)	CRF	314	exercise	increased CRF
Courneya, K.S. et al., 2009 [59]	lymphoma	CRF	122	aerobic exercise training	increased CRF
Speck, R.M. et al., 2010 [60]	mixed (meta analysis) cancer patients	CRF	6838	physical activity	increased CRF
Jones, L.W. et al., 2011 [61]	mixed (meta analysis) cancer patients	CRF	571	supervised exercise training	increase in VO_2peak_
Courneya, K.S. et al., 2007 [62]	breast cancer	CRF + quality of life	242	aerobic and resistance exercise	increased CRF, no change in quality of life

**Table 3:** Cardiac exercise rehabilitation effects on cardiovascular aspects of cancer patients during therapy with cardiotoxic drugs. Systemic vascular resistance: SVR; ejection fraction: EF; left ventricle: LV; B-type natriuretic peptide: BNP; N-terminal prohormone of brain natriuretic peptide: NT-proBNP.

Hemodynamics	SVR [75] 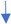 Cardiac output [76] 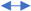 Blood Pressure [75] 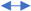
Echocardiographic	EF [75,76,77] 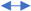 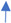 LV remodeling [75] 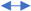 LV longitudinal strain [75,76] 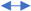 E/A ratio [75] 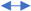 LV mass [75] 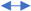 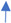
Biomarkers	Troponin [76] 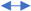 NT-proBNP [77] 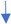
Cardiorespiratory function	VO_2peak_ [76] 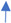 Muscle strength [50] 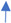 Fatigue [50] 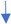
